# Probiotics impact the antibiotic resistance gene reservoir along the human GI tract in a person-specific and antibiotic-dependent manner

**DOI:** 10.1038/s41564-021-00920-0

**Published:** 2021-07-05

**Authors:** Emmanuel Montassier, Rafael Valdés-Mas, Eric Batard, Niv Zmora, Mally Dori-Bachash, Jotham Suez, Eran Elinav

**Affiliations:** 1 Microbiota Hosts Antibiotics and Bacterial Resistances, Université de Nantes, Nantes, France; 2 Department of Emergency Medicine, Centre Hospitalier Universitaire de Nantes, Nantes, France; 3 Université de Nantes, EA3826 Thérapeutiques Anti-Infectieuses, Institut de Recherche en Santé 2 Nantes Biotech, Nantes, France; 4 Immunology Department, Weizmann Institute of Science, Rehovot, Israel; 5 Research Center for Digestive Tract and Liver Diseases, Tel Aviv Sourasky Medical Center, Sackler Faculty of Medicine, Tel Aviv University, Tel Aviv, Israel; 6 Internal Medicine Department, Tel Aviv Sourasky Medical Center, Tel Aviv, Israel; 7 Division of Cancer-Microbiome Research, German Cancer Research Center, Heidelberg, Germany; 8 Present Address: Department of Molecular Microbiology and Immunology, Johns Hopkins Bloomberg School of Public Health, Baltimore, MD USA

**Keywords:** Dysbiosis, Microbiome

## Abstract

Antimicrobial resistance poses a substantial threat to human health. The gut microbiome is considered a reservoir for potential spread of resistance genes from commensals to pathogens, termed the gut resistome. The impact of probiotics, commonly consumed by many in health or in conjunction with the administration of antibiotics, on the gut resistome is elusive. Reanalysis of gut metagenomes from healthy antibiotics-naïve humans supplemented with an 11-probiotic-strain preparation, allowing direct assessment of the gut resistome in situ along the gastrointestinal (GI) tract, demonstrated that probiotics reduce the number of antibiotic resistance genes exclusively in the gut of colonization-permissive individuals. In mice and in a separate cohort of humans, a course of antibiotics resulted in expansion of the lower GI tract resistome, which was mitigated by autologous faecal microbiome transplantation or during spontaneous recovery. In contrast, probiotics further exacerbated resistome expansion in the GI mucosa by supporting the bloom of strains carrying vancomycin resistance genes but not resistance genes encoded by the probiotic strains. Importantly, the aforementioned effects were not reflected in stool samples, highlighting the importance of direct sampling to analyse the effect of probiotics and antibiotics on the gut resistome. Analysing antibiotic resistance gene content in additional published clinical trials with probiotics further highlighted the importance of person-specific metagenomics-based profiling of the gut resistome using direct sampling. Collectively, these findings suggest opposing person-specific and antibiotic-dependent effects of probiotics on the resistome, whose contribution to the spread of antimicrobial resistance genes along the human GI tract merit further studies.

## Abstract (web summary)

Direct gut sampling shows that probiotics reduce the number of antibiotic resistance genes in the gut of colonization-permissive and antibiotic-naïve individuals. However, when given after antibiotic treatment, probiotics can expand the gut resistome via a bloom of indigenous strains carrying vancomycin resistance genes, rather than antibiotic resistance genes carried by the probiotics themselves.

## Main

Antimicrobial resistance (AMR) constitutes a prominent health threat, accounting for an annual death toll of 700,000, which is projected to increase to up to 10 million fatalities worldwide by 2050 (ref. ^1^). The gut microbiome serves as a reservoir of antibiotic resistance genes (ARGs)^2–4^, which could potentially transfer horizontally to pathogens and contribute to the emergence of drug-resistant bacteria^5^. Understanding the factors that shape the human gut resistome and devising means to circumvent resistome expansion are likely to facilitate the fight against AMR. A prominent contributor to resistome expansion is the use of antibiotics^6,7^. Additionally, transfer of ARGs from pathogens to commensals has been demonstrated experimentally^8–12^, in patients^13–16^ and through the food chain^6^. In this context, probiotics have been hailed as means for restoring microbiome balance after perturbation by antibiotics and, consequently, prevent resistome expansion^17^. Nonetheless, ARGs have been identified in commercial probiotic products^18^ and in genomes of common probiotic supplement species^19^, raising concerns that at least some of these ARGs can transfer to commensals and pathogens^20^. Currently, the extent to which probiotics modulate the microbiome is contested^21,22^ and their effect on the resistome is unclear. In antibiotic-treated adults^23^ or infants^24^, probiotics did not demonstrate superior resistome mitigation compared to placebo or no probiotics. However, the resilience of the adult microbiome to the perturbation and multiple baseline differences between the infant treatment groups complicate the interpretation of these findings. A potential caveat in current studies is the exclusive reliance on stool samples, which only partly reflect the gastrointestinal (GI) microbiome and are oblivious to interindividual differences in GI probiotic colonization^25,26^. In this study, we performed an analysis of an existing metagenome dataset to characterize the human gut resistome in situ in endoscopy samples with paired stool samples and characterized the effects of antibiotics, probiotics and autologous faecal microbiome transplantation on the ARG reservoir in multiple cohorts^26,27^. We demonstrated that a commercially available 11-strain probiotic mix can reduce the number of ARGs in colonization-permissive, antibiotic-naïve individuals. In contrast, after a course of antibiotics, these probiotic strains exacerbated the antibiotic-mediated resistome expansion in the lower GI tract mucosa but did not contribute to the increase in ARGs from their own repertoire.

## Results

### Stool samples do not accurately reflect the gut resistome

**Fig. 1:**
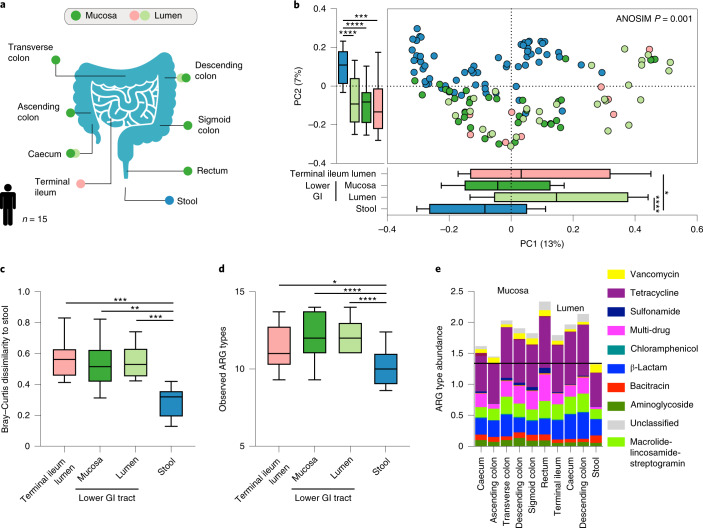
Stool samples do not represent the GI resistome. Fifteen men and women provided stool samples and underwent a session of colonoscopy after 7 d of providing stool samples, during which luminal aspirates were collected from the terminal ileum, caecum and descending colon; mucosal brushes were collected from the caecum, ascending colon, transverse colon, descending colon, sigmoid colon and rectum. Metagenomic sequences were subsampled to 2 M of reads, resulting in 65 stool (blue), 29 lower GI tract luminal aspirates (light green), 12 terminal ileum luminal aspirates (peach) and 32 mucosal brush samples (dark green) analysed using ARG-OAP v.2.0 to identify and quantify ARG ‘subtypes’. **a**, Sampled GI tract regions. **b**, Bray–Curtis-based beta diversity of stool and endoscopic samples based on ARG subtypes. PC1 stool versus terminal ileum lumen *P* = 0.041, stool versus lower GI tract lumen *P* < 0.0001; PC2 stool versus terminal ileum lumen *P* = 0.0001, stool versus lower GI tract mucosa *P* < 0.0001, stool versus lower GI tract lumen *P* < 0.0001. **c**, Bray–Curtis dissimilarity to stool in samples from the terminal ileum lumen (*P* = 0.0003), lower GI tract mucosa (*P* = 0.003) and lower GI tract lumen (*P* = 0.0002) based on ARG subtypes. **d**, The observed ARG ‘types’ (alpha diversity) are significantly lower in stool compared to the terminal ileum lumen (*P* = 0.0467), lower GI tract mucosa (*P* < 0.0001) and lower GI tract lumen (*P* < 0.0001). **e**, Abundance of antibiotic resistance ‘types’ per region. *P < 0.05, **P < 0.01, ***P < 0.001, ****P < 0.0001, Kruskal–Wallis and Dunn’s tests (all panels). The horizontal lines represent the median and the whiskers represent the 10–90 percentiles. Source data

**Extended Data Fig. 1:**
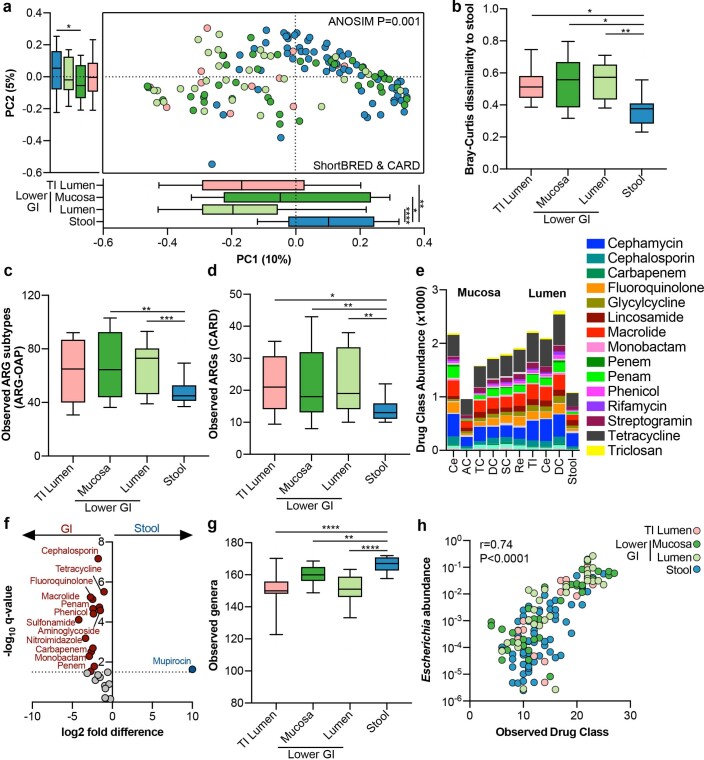
Stool samples do not represent the gastrointestinal resistome in antibiotics-naïve and treated individuals. Fifteen men and women provided stool samples, and underwent a session of colonoscopy, during which luminal aspirates were collected from the terminal ileum, cecum, and descending colon; and mucosal brushes were collected from the cecum, ascending colon, transverse colon, descending colon, sigmoid colon, and rectum. As a validation to the analysis in Fig. 1, metagenomic sequences were subsampled to 1.5 M reads, resulting in 66 stool (blue), 29 lower GI luminal aspirates (light green), 14 terminal ileum luminal aspirates (peach), and 39 lower GI mucosal brush (dark green) samples analyzed using ShortBRED & CARD for identifying and quantifying ARGs. **a**, Bray-Curtis dissimilarity of stool and endoscopic samples, based on ARG families. PC1 stool vs. TI lumen P = 0.002,, stool vs. lower GI mucosa P = 0.025, stool vs. lower GI lumen P < 0.0001; PC2 stool vs. lower GI mucosa P = 0.0175. **b**, Bray-Curtis dissimilarity to stool in samples from the TI lumen (P = 0.0139), lower GI mucosa (P = 0.0187), and lower GI lumen (P = 0.0027), based on ARG families. **c**, Observed ARGs ‘subtypes’ (ARG-OAP v2.0, alpha diversity) is lower in stool compared to the lower GI mucosa (P = 0.0012) and lower GI lumen (P = 0.0008). **d**, Observed ARGs using CARD & ShortBRED is lower in stool compared to TI lumen (P = 0.0213), lower GI mucosa (P = 0.0027), lower GI lumen (P = 0.0016). **e**, Abundance of drug classes per region. **f**, Drug classes significantly overrepresented in the GI (red) or stool (blue). Colored circles represent P < 0.05 (FDR-corrected two-sided Mann-Whitney). **g**, Observed genera (alpha diversity), rarefied to 2 M reads, is higher in stool compared to TI lumen (P < 0.0001), lower GI mucosa (P = 0.0015), and lower GI lumen (P < 0.0001). **h**, Spearman correlation (P < 0.0001) of *Escherichia* abundance with observed drug class. *, P < 0.05; **, P < 0.01; ***, P < 0.001; ****, P < 0.0001, Kruskal-Wallis & Dunn’s. Horizontal lines represent the median, whiskers 10-90 percentiles. GI, gastrointestinal tract; TI, terminal ileum. Source data

Since we recently reported that microbiome functional gene content differs between stool and endoscopy-collected GI samples, we sought to examine whether this distinction applies specifically to the gut resistome. We reanalysed data from 15 healthy human participants who underwent a colonoscopy while concomitantly providing stool samples (Fig. 1a) and characterized their resistome using two approaches: the Antibiotic Resistance Gene Online Analysis Pipeline (ARGs-OAP) v.2.0 (ref. ^28^) and quantification of translated ARG abundance using the Comprehensive Antibiotic Resistance Database (CARD)^29^ and ShortBRED^30^. After even subsampling, Bray–Curtis dissimilarities readily separated the resistome of stool and endoscopic samples using both ARG-OAP (subsampled to 2 M; Fig. 1b; analysis of similarities (ANOSIM) *P* = 0.001) as well as ShortBRED and CARD (subsampled to 1.5 M; Extended Data Fig. 1a; ANOSIM *P* = 0.001); samples from both the mucosa and even the lumen of the lower GI tract clustered separately from stool samples (ARG-OAP Kruskal–Wallis and Dunn’s tests *P* < 0.01, Fig. 1c; ShortBRED and CARD *P* < 0.05, Extended Data Fig. 1b). The number of observed ARGs (alpha diversity) was significantly lower in stool samples (Kruskal–Wallis *P* < 0.0001 based on ARG types, Fig. 1d; *P* = 0.0002 based on ARGs, Extended Data Fig. 1d), stemming from lower abundance of all observed types in stool samples rather than under-representation of specific ARGs (types in Fig. 1e; drug classes in Extended Data Fig. 1e,f).

In contrast to resistome diversity, stool samples were characterized by the highest taxonomic diversity (Kruskal–Wallis test *P* < 0.0001 versus lower GI tract lumen and terminal ileum lumen, *P* = 0.0015 versus lower GI tract mucosa; Extended Data Fig. 1g). Thus, the under-representation of the resistome in stool samples was not a result of lower taxonomic diversity but rather due to under-representation of specific species, mostly in the *Escherichia* genus (Extended Data Fig. 1h). Collectively, stool samples under-represented the GI tract resistome, necessitating the use of endoscopic samples for proper assessment of the effect of probiotics on the gut resistome.

### Probiotic colonization is associated with a reduced ARG load in endoscopic samples

**Fig. 2:**
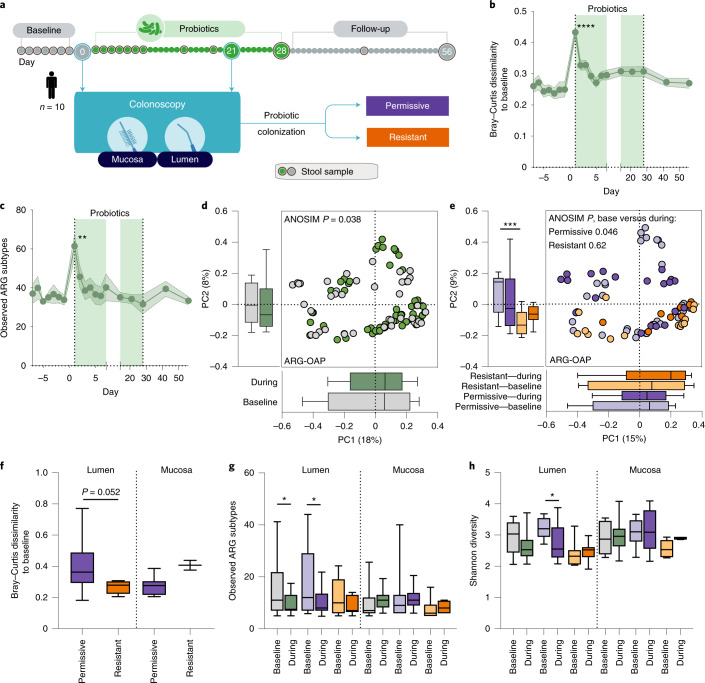
Probiotic-associated reduction in gut resistome is person-specific. Ten men and women provided stool samples before, after and during 28 d of supplementation with a commercial probiotic supplement; two colonoscopies were performed immediately before supplementation started and on day 21 of supplementation. Metagenomic sequences were analysed using ARG-OAP v.2.0 for the identification of ARGs, subsampled to 2 M of reads and normalized by 16S. **a**, Experimental design. Individuals were defined as colonization-permissive if they had a statistically significant increase in probiotic load in their lower GI tract mucosa samples according to species-specific quantitative PCR amplification^26^. **b**, Bray–Curtis dissimilarity (ARG subtypes) of stool samples to all baseline samples of each individual. The light green shade indicates the supplementation period. Day 1 of supplementation versus baseline *P* < 0.0001. **c**, Observed ARG subtypes in stool over time (*P* = 0.0031). **d**, Bray–Curtis dissimilarity of ARGs (‘types’) in all lower GI tract endoscopic samples (luminal aspirates and mucosal brushes) collected before (grey) or during supplementation (day 21, green). **e**, Same as **d** but based on ARG subtypes and colour-coded according to probiotic colonization permissiveness (purple, *n* = 6) or resistance (orange, *n* = 4) and time point (before, light; during, dark). PC2 permissive versus resistant baseline *P* = 0.0004. **f**, Per-person Bray–Curtis dissimilarity to baseline calculated in all participants or in the two subsets based on ARG subtypes. Lumen *P* = 0.052. **g**,**h**, Alpha diversity measurements (**g**), observed ARGs (subtypes) or Shannon diversity index in endoscopic samples (**h**) of permissive and resistant individuals, compared either to the baseline of each subset or between subsets. In **g**, lumen, all samples baseline versus during *P* = 0.035, permissive baseline versus during *P* = 0.0223. In **h**, lumen, permissive baseline versus during *P* = 0.0226. *P < 0.05; **P < 0.01; ***P < 0.001; ****P < 0.0001. Two-way ANOVA and Dunnett’s (**a**,**b**) or Sidak’s test (**g**,**h**) or two-sided Mann–Whitney *U*-test (all the rest). The horizontal lines represent the median, the symbols represent the mean, the error bands represent the s.e.m. (**b**,**c**) and the whiskers represent the 10–90 percentiles (**d**–**h**). Source data

**Extended Data Fig. 2:**
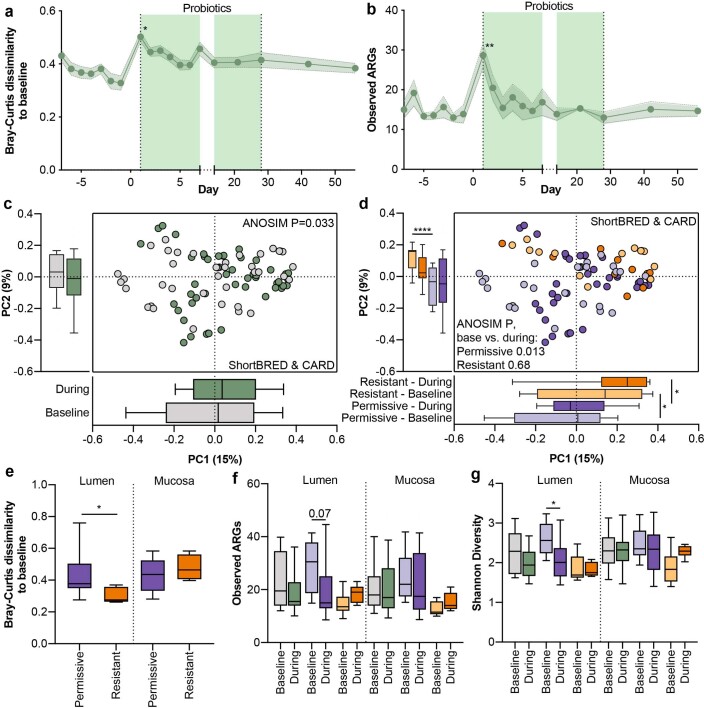
Probiotics-associated reduction in gut resistome is person-specific. Ten men and women provided stool samples before, after, and during 28 days of supplementation with a commercial probiotic supplement; two colonoscopies were performed, immediately before supplementation started, and on day 21 of supplementation. Metagenomic sequences were analyzed using ShortBRED & CARD for identification of ARGs, subsampled to 1.5 M reads. Results are based on ARGs. **a**, Bray-Curtis dissimilarity of stool samples to all baseline samples of each individual. The light green shade indicates the supplementation period. Day 1 of supplementation vs. baseline P = 0.0155. **b**, Observed ARGs in stool over time (P = 0.0014). **c**, Bray-Curtis dissimilarity of ARGs in all lower GI endoscopic samples (luminal aspirates and mucosal brushes) collected before (grey) or during supplementation (day 21, green). **d**, Same as C but color-coded according to probiotics colonization permissiveness (purple, N = 6) or resistance (orange, N = 4) and timepoint (before, light; during, dark). PC1 baseline P = 0.0472, during P = 0.01; PC2 baseline P < 0.0001. **e**, Per-person Bray-Curtis dissimilarity to baseline calculated in all participants or in the two subsets (P = 0.038). **f-g**, Alpha diversity measurements (**f**) observed ARGs or **g**, Shannon diversity in endoscopic samples of permissive and resistant individuals, compared either to the baseline of each subset or between subsets. In G, lumen, permissive P = 0.0188. *, P < 0.05; **, P < 0.01; ****, P < 0.0001, Two-Way ANOVA & Dunnett (**a**-**b**) or Sidak (**f**-**g**), or two-sided Mann-Whitney (all the rest). Horizontal lines represent the median, symbols represent mean, error bands SEM (A-B), whiskers 10-90 percentiles. Source data

To determine the effect of probiotics on the gut resistome, we analysed the metagenomic sequences from 10 healthy individuals who underwent two colonoscopy sessions before and during supplementation (day 21) with a commercially available oral probiotic supplement (Bio-25, containing 11 probiotic strains from the *Lactobacillus*, *Bifidobacterium*, *Streptococcus* and *Lactococcus* genera; Fig. 2a). The effect of probiotics on the stool resistome was restricted to the first day of supplementation as reflected in ARG-based beta diversity (two-way analysis of variance (ANOVA) and Dunnett’s test *P* < 0.0001, ARG-OAP, Fig. 2b; P < 0.05, ShortBRED and CARD, Extended Data Fig. 2a) as well as a transient increase in the number of observed ARGs (subtypes in ARG-OAP *P* = 0.0031, Fig. 2c; ARGs in ShortBRED and CARD *P* = 0.0014, Extended Data Fig. 2b). In contrast, probiotics significantly increased the resistome configuration dissimilarity to pre-supplementation baseline in endoscopic samples based on types (ARG-OAP ANOSIM *P* = 0.038; Fig. 2d) or ARGs (ShortBRED and CARD *P* = 0.033; Extended Data Fig. 2c). We previously reported that a subset of individuals resist probiotic colonization in the GI tract mucosa^26^ and even exclude these bacteria from the gut lumen^31^; therefore, we sought to determine whether probiotic colonization underlies their effect on the resistome. Interestingly, the resistomes of permissive and resistant individuals were different at baseline (ARG-OAP ANOSIM *P* = 0.029, Mann–Whitney *U*-test *P* = 0.0004 on PC2, Fig. 2e; ShortBRED and CARD ANOSIM *P* = 0.021, *P* = 0.047 PC1, *P* < 0.0001 PC2, Extended Data Fig. 2d). After probiotic supplementation, we observed a significant increase in resistome dissimilarity to pre-supplementation baseline in colonization-permissive (ARG-OAP ANOSIM *P* = 0.046, Fig. 2e; ShortBRED and CARD *P* = 0.013, Extended Data Fig. 2d) but not colonization-resistant individuals (*P* = 0.62, *P* = 0.68) attributed to the intestinal lumen (ARG-OAP *P* = 0.052, Fig. 2f; ShortBRED and CARD *P* = 0.038, Extended Data Fig. 2e). This increased dissimilarity was associated with a reduction in resistome load (ARG-OAP permissive lumen *P* = 0.022, Fig. 2g; ShortBRED and CARD *P* = 0.07, Extended Data Fig. 2f) and diversity (ARG-OAP *P* = 0.023, Fig. 2h; ShortBRED and CARD *P* = 0.019, Extended Data Fig. 2g), which was restricted to luminal samples from colonization-permissive individuals. This analysis suggested that probiotics can reduce the burden of ARGs in the intestines of antibiotic-naïve individuals in a probiotic colonization-dependent manner and that this beneficial effect cannot be inferred from stool samples.

### Antibiotics expand the resistome in the lower GI tract

**Fig. 3:**
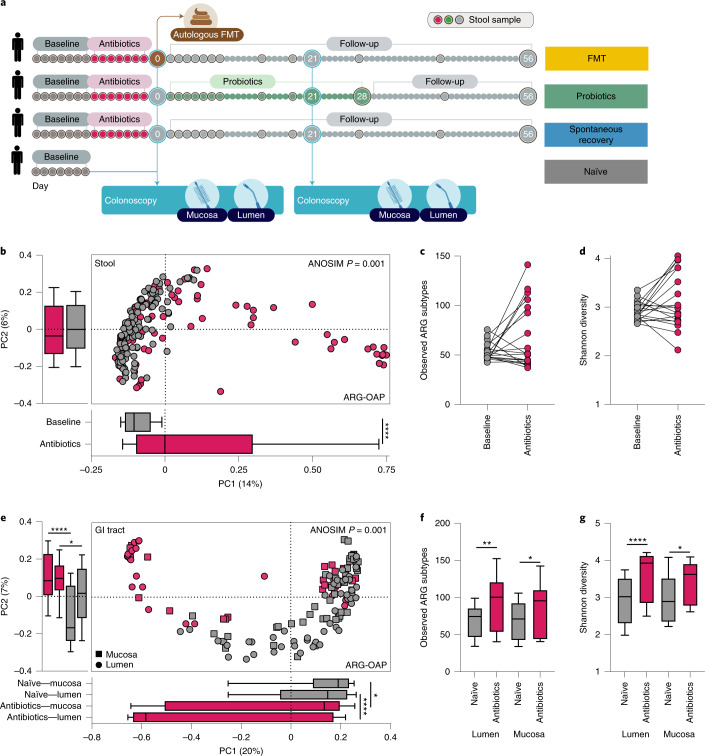
Antibiotics expand the resistome in the lower GI tract lumen. **a**, Experimental design of antibiotics treatment and follow-up arms. **b**–**d**, Metagenomic sequences, subsampled to 2 M of reads, were analysed using ARG-OAP v.2.0 to identify ARGs and normalized by 16S. The results are based on ARG subtypes. Stool samples were collected from 21 individuals for 7 d before (grey) and 7 d during (magenta) a course of ciprofloxacin and metronidazole. **b**, Bray–Curtis dissimilarities (*P* < 0.0001). **c**,**d**, Paired comparison of alpha diversity (**c**) observed ARG subtypes or Shannon diversity index (**d**). Each point represents the average of all baseline or antibiotic days for each individual. **e**–**g**, The 21 participants underwent endoscopy immediately after 7 d of antibiotics (magenta). We compared their resistome to individuals undergoing endoscopy without any treatment (*n* = 15, grey). **e**, Bray–Curtis dissimilarities. PC1 lumen *P* < 0.0001, mucosa *P* = 0.0196; PC2 lumen *P* < 0.0001, mucosa *P* = 0.044. **f**,**g**, Alpha diversity (**f**) observed ARG subtypes (lumen *P* = 0.0011, mucosa *P* = 0.034) or Shannon diversity index (**g**) (lumen *P* < 0.0001, mucosa *P* = 0.0143). *P < 0.05; **P < 0.01; ****P < 0.0001. Two-sided Mann–Whitney *U*-test. The horizontal lines represent the median, the symbols represent the mean and the whiskers represent the 10–90 percentiles. Source data

**Extended Data Fig. 3:**
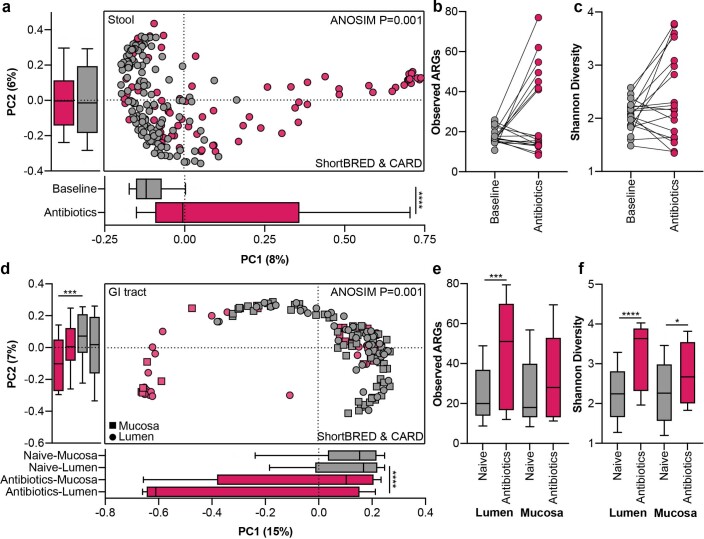
Antibiotics expand the resistome in the lower GI lumen. **a**, Experimental design of antibiotics treatment and follow-up arms. Validation analysis with ShortBRED-CARD and subsampled to 1.5 M. **a-c**, Stool samples were collected from 21 individuals for seven days before (grey) and seven days during (magenta) a course of ciprofloxacin and metronidazole. **a**, Bray-Curtis dissimilarities, based on ARG families (PC1 P < 0.0001). **b-c**, Paired comparison of alpha diversity (**b**) observed ARGs or **c**, Shannon diversity. In B-C, each point represents the average of all baseline or antibiotics days for each individual. **d-f**, The 21 participants underwent endoscopy immediately after 7 days of antibiotics (magenta). We compared their resistome to individuals undergoing endoscopy without any treatment (N = 15, grey). **d**, Bray-Curtis dissimilarities of ARG families (PC1 lumen P < 0.0001; PC2 lumen P = 0.0007). **f-g**, Alpha diversity (**e**) observed ARGs (P = 0.0005) or **f**, Shannon diversity (lumen P < 0.0001; mucosa P = 0.0191). *, P < 0.05; ***, P < 0.001; ****, P < 0.0001, two-sided Mann-Whitney. Horizontal lines represent the median, symbols mean, whiskers 10-90 percentiles. Source data

Importantly, the effect of antibiotics on the gut resistome was previously reported using stool samples but not through direct sampling. Therefore, we next analysed the resistome of 21 healthy (that is, no active infection) adults who received antibiotics for 7 d (500 mg of oral ciprofloxacin twice daily and 500 mg of oral metronidazole three times daily)^27^. These individuals provided stool samples before and during antibiotics and underwent a colonoscopy after 7 d of treatment (Fig. 3a). Antibiotics significantly increased the resistome dissimilarity to the pre-antibiotic baseline (ANOSIM *P* = 0.001, Fig. 3b) but had no conclusive effect on the number of observed ARG subtypes (Wilcoxon test *P* = 0.37, Fig. 3c) or Shannon diversity index (Wilcoxon test *P* = 0.33, Fig. 3d) in stool samples due to interindividual heterogeneity. Samples from the lower GI tract of the aforementioned 21 individuals were significantly different from those of 24 antibiotic-naïve individuals (ANOSIM *P* = 0.001, Fig. 3e). Antibiotics significantly elevated both the number of observed ARG subtypes (Kruskal–Wallis test *P* = 0.0019, Fig. 3f) as well as Shannon diversity index (*P* < 0.0001, Fig. 3g) in the lower GI tract. The aforementioned observations obtained with ARG-OAP were highly similar with CARD and ShortBRED (Extended Data Fig. 3). Thus, stool samples were insufficient to assess antibiotic-induced gut resistome expansion in situ.

### After antibiotic treatment, probiotics are associated with increased ARG content compared to autologous faecal microbiome transplantation and spontaneous recovery in endoscopic samples

**Fig. 4:**
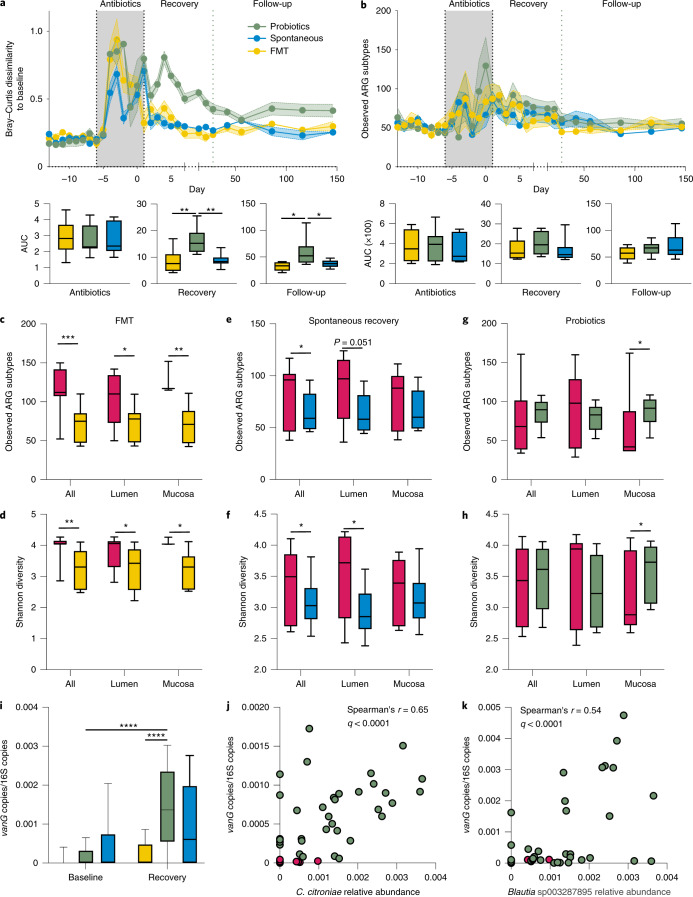
Probiotics expand the resistome in the GI tract mucosa after antibiotics. **a**,**b**, Longitudinal follow-up of resistome (analysed using ARG-OAP v.2.0, subsampled to 2 M of reads, normalized by 16S) in stool samples of 21 individuals before and during antibiotics (magenta) and then through 3 post-antibiotics recovery groups: spontaneous recovery (blue, *n* = 7), autologous FMT performed on day 0 (yellow, *n* = 6) or probiotic supplementation between days 0 and 28 (green, *n* = 8). (The green horizontal line denotes the end of the supplementation period.) **a**, Bray–Curtis dissimilarities and incremental area under the curve (AUC) to each individual’s baseline (all baseline samples), based on ARG subtypes. Recovery probiotics versus spontaneous *P* = 0.0063, probiotics versus FMT *P* = 0.0063; follow-up probiotics versus spontaneous *P* = 0.0264, probiotics versus FMT *P* = 0.0238. **b**, Same as **a** but observed ARG subtypes. AUC (×100) values were divided by 100 for presentation purposes. **c**–**h**, Comparison of ARG-based (subtypes) alpha diversity metrics for observed ARGs (**c**,**e**,**g**) or Shannon diversity index (**d**,**f**,**h**) in lower GI tract samples of participants in the FMT (**c**,**d**), spontaneous recovery (**e**,**f**) or probiotics (**g**,**h**) group. **c**, FMT all samples *P* = 0.0003, lumen *P* = 0.024, mucosa *P* = 0.0026. **d**, FMT all samples *P* = 0.0024, lumen *P* = 0.031, mucosa *P* = 0.04. **e**, Spontaneous all samples *P* = 0.044. **f**, Spontaneous all samples *P* = 0.029, lumen *P* = 0.0446. **g**, Probiotics mucosa *P* = 0.015. **h**, Probiotics mucosa *P* = 0.038. **i**, Abundance of the *vanG* gene in the endoscopic samples of each group after antibiotics and after 21 d of recovery. Probiotics recovery versus antibiotics *P* < 0.0001, probiotics versus FMT *P* < 0.0001. **j**,**k**, Bacterial species (*C. citroniae*, **j**; *Blautia* sp003287895, **k**) significantly (*P* < 0.0001) correlated (Spearman) with *vanG* abundance in endoscopic samples. *P < 0.05, **P < 0.01, ***P < 0.001, ****P < 0.0001. One-way ANOVA and Sidak’s test (**a**), two-way ANOVA and Sidak’s (**i**) or Dunnett’s test (**i**) or two-sided Mann–Whitney *U*-test (all the rest). The horizontal lines represent the median, the symbols represent the mean (**a**,**b** main panels), the error bands represent the s.e.m. (**a**,**b** main panels) and the whiskers represent the 10–90 percentiles. Source data

**Extended Data Fig. 4:**
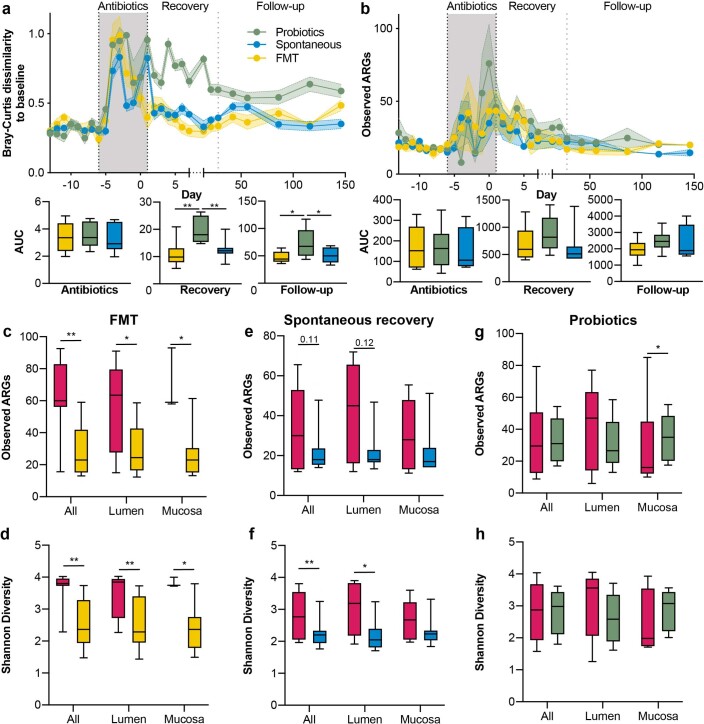
Probiotics expand the resistome in the GI mucosa after antibiotics. Longitudinal follow-up of resistome (analyzed using ShortBRED & CARD, subsampled to 2 M reads) in stool samples of 21 individuals before and during antibiotics (magenta), and then through 3 post-antibiotics recovery groups: spontaneous recovery (blue, N = 7), autologous fecal microbiome transplantation (FMT) performed on day 0 (yellow, N = 6), or probiotics supplementation between days 0-28 (green, N = 8) (green horizontal line denotes end of supplementation period). Results based on ARGs. **a**, Bray-Curtis dissimilarities and incremental area under the curve to each individual’s baseline. Recovery probiotics vs. spontaneous P = 0.0088, probiotics vs. FMT P = 0.0055; follow-up probiotics vs. spontaneous P = 0.0426, probiotics vs. FMT P = 0.0385. **b**, Same as A but observed ARGs. **c-h**, Comparison of alpha diversity metrics observed ARGs (**c**, **e**, **g**) or Shannon Diversity (**d**, **f**, **h**) in lower GI samples of participants (analyzed using ShortBRED & CARD, subsampled to 1.5 M) in the FMT (**c**-**d**), spontaneous recovery (**e**-**f**), or probiotics (**g**-**h**) group. **c**, FMT all samples P = 0.001, Lumen P = 0.0329, Mucosa P = 0.0193. **d**, FMT all samples P = 0.001, Lumen P = 0.0041, Mucosa P = 0.0281. **f**, Spontaneous all samples P = 0.0092, lumen P = 0.0246. **g**, Probiotics mucosa P = 0.0388. *, P < 0.05, **, P < 0.01, One-Way ANOVA & Sidak (**a**), or two-sided Mann-Whitney (all the rest). Horizontal lines represent the median, symbols mean (a-b main panels), error bands SEM (**a**-**b** main panels), whiskers 10-90 percentiles. Source data

The effect of probiotics on antibiotic-associated resistome expansion is currently elusive. Therefore, we analysed the resistome in the aforementioned 21 individuals when assigning them to three post-antibiotics recovery arms (Methods and Fig. 3a): probiotics (*n* = 8); autologous faecal microbiome transplantation (FMT) (*n* = 6); or spontaneous recovery (*n* = 7)^27^. In stool samples, antibiotics increased the dissimilarity to baseline resistome in individuals in all groups, with the resistome of the probiotics group being the slowest to recover (Fig. 4a). This potentially stemmed from a sustained expansion of resistome in the probiotics group (Fig. 4b); however, this did not reach statistical significance. Direct gut sampling after 21 d of recovery revealed that autologous FMT was the most effective for reverting the antibiotic-associated resistome expansion in the lower GI tract (observed ARG subtypes, Mann–Whitney *U*-test *P* = 0.0003; lumen *P* = 0.024; mucosa *P* = 0.0026, Fig. 4c; Shannon *P* = 0.0024; lumen *P* = 0.031; mucosa *P* = 0.04, Fig. 4d). Spontaneous recovery also reverted resistome expansion (observed ARG subtypes, *P* = 0.044, Fig. 4e; Shannon *P* = 0.029, Fig. 4f), mainly in the lumen. In contrast, probiotics did not revert resistome expansion (observed ARG subtypes *P* = 0.27; lumen *P* = 0.25, Fig. 4g; Shannon *P* = 0.71; lumen *P* = 0.25, Fig. 4h) but rather further expanded the number of ARG subtypes in the gut mucosa (*P* = 0.015, Fig. 4g; Shannon *P* = 0.038, Fig. 4h). These observations were validated by ShortBRED and CARD (Extended Data Fig. 4). Species-based Bray–Curtis dissimilarity to antibiotic-naïve samples was positively correlated with ARG-based beta diversity (Spearman’s *r* = 0.45, *P* < 0.0001) and species-based alpha diversity was inversely weakly correlated with resistome expansion (*r* = 0.2, *P* < 0.0001), suggesting that a greater inhibitory effect on microbiome recovery from antibiotics (as observed in the probiotics group) results in greater resistome expansion.

**Extended Data Fig. 5:**
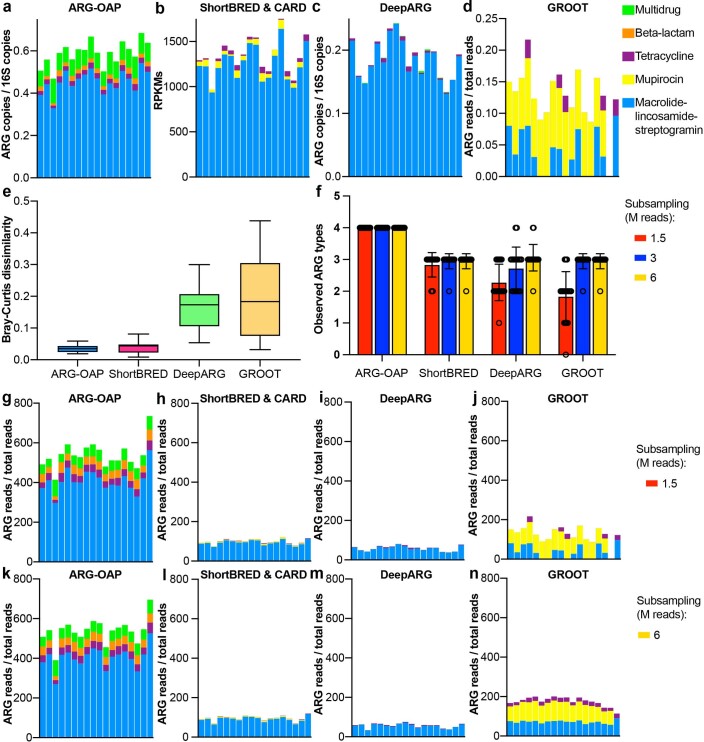
Comparison of methods for detecting ARGs in metagenomic samples. Whole-genome shotgun metagenomics sequencing was performed on eighteen pills of the Bio25 probiotic supplement from different batches. We quantified the abundance of ARG types (detected by genome assembly, see Methods) using 4 ARG pipelines and different subsampling sizes (1.5 M, 3 M and 6 M reads): **a**, ARG_OAP v2.0, using Hidden Markov Models and the SARG 2.0 database. **b**, shortBRED, combined with the CARD database, and based on unique peptide sequences for each ARG family. **c**, DeepARG-DB. **d**, variation graph method, GROOT, in combination with the CARD database. **e**, Bray-Curtis dissimilarity to baseline (1.5 M) between the 18 pills when analyzed with the different pipelines and subsampled to 6 M. **f**, Observed ARG types in each method and subsampling depth. **g-j**, Same as A-D but ARG abundances are reported as ARG read count per total reads; subsampled to 1.5 M reads. **k-n**, Same as G-J but subsampled to 3 M reads. Horizontal lines represent the median, whiskers 10-90 percentiles, error bars SEM. RPKM, reads per kilobase of reference sequence per million sample reads. Source data

Since we identified an association between probiotic supplementation and resistome expansion in the intestinal mucosa, we sought to determine whether the source of these ARGs is the supplemented probiotic strains. To that purpose, we first defined resistome content by genome assembly and ARG annotation of three paired-end sequenced tablets (Methods). We then quantified the identified ARGs in 18 Bio-25 tablets from different batches using ARG-OAP and ShortBRED and CARD, subsampled to 1.5 M of reads, the same threshold applied to the intestinal samples. The majority of ARG types found in the tablets according to both ARG-OAP (Extended Data Fig. 5a) and ShortBRED and CARD (Extended Data Fig. 5b) belonged to the macrolide-lincosamide-streptogramin ARG type, followed by tetracycline resistance. However, ARGs belonging to multi-drug resistance or the β-lactam types were only identified in ARG-OAP and ARGs belonging to the mupirocin type were only found by ShortBRED and CARD. To potentially resolve this discrepancy, we applied two additional pipelines to detect ARGs in the tablets: DeepARG^32^ (Extended Data Fig. 5c) and GROOT^33^ (Extended Data Fig. 5d) in combination with CARD. Although these two additional methods also reported ARGs from the macrolide and tetracycline classes in the tablets, DeepARG found multi-drug ARGs (similar to ARG-OAP), and GROOT-mupirocin ARGs (similar to ShortBRED). Notably, between-tablet heterogeneity was considerably higher for DeepARG and GROOT and (to a lesser extent) for ShortBRED, compared to ARG-OAP (Extended Data Fig. 5e). When analysed this with three different subsampling depths (1.5, 3 and 6 M of reads); DeepARG, GROOT and ShortBRED demonstrated high heterogeneity and reduced diversity in lower sequencing depths. In contrast, ARG-OAP identified the same number of ARG types at all sequencing depths (Extended Data Fig. 5f). These discrepancies (Extended Data Fig. 5g–n) likely represent a trade-off between specificity and sensitivity (Methods); thus, cross-validation may be required for resistome profiling.

Notably, using ARG-OAP, we searched for genes that were significantly elevated in the post-antibiotics probiotics group, compared to spontaneous recovery and FMT. *vanG*, encoding for vancomycin resistance, was significantly elevated in the probiotics group compared to baseline or to the group recovering with FMT (multiple-testing corrected Mann–Whitney *U*-test *q* < 0.0001, Fig. 4i). Since we could not detect vancomycin resistance genes in the supplemented tablet (Extended Data Fig. 5), we next asked whether the source of *vanG* is the endogenous microbiome. Notably, while probiotics inhibited the recovery of microbial diversity, they promoted the expansion of a limited number of species that were significantly less abundant in the FMT or spontaneous recovery groups. Four of these species were significantly correlated with *vanG* abundance: *Clostridium citroniae* (Fig. 4j), *Clostridium leptum*, an unnamed *Blautia* sp. (Fig. 4k) and *Romboutsia timonensis*. Thus, the inhibitory effect of probiotics on microbiome recovery from antibiotics allowed for the expansion of species that likely carry the expanding clinically relevant ARGs.

### Probiotics are associated with post-antibiotic resistome expansion in mice

**Fig. 5:**
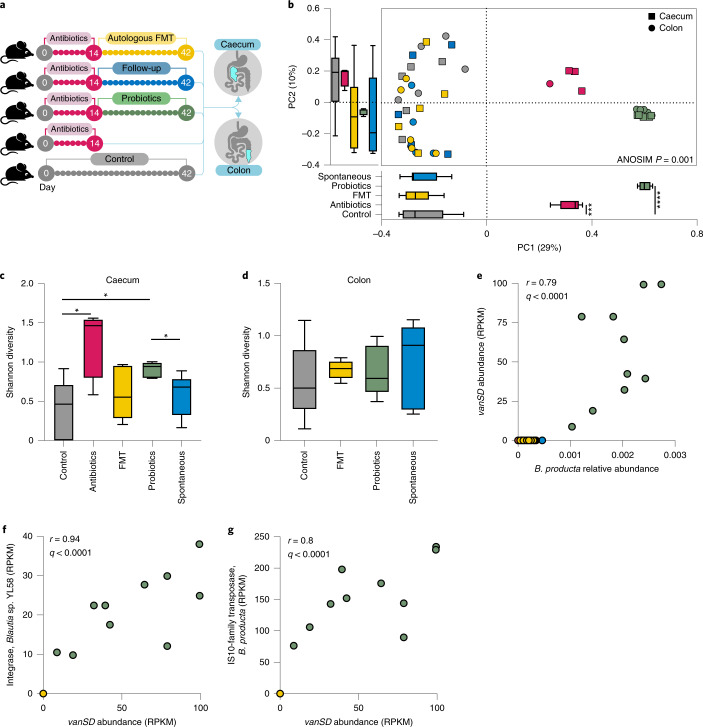
Probiotics expand the GI tract resistome in antibiotic-treated mice. **a**, Experimental design. Wild-type adult (10-week-old) male C57BL/6J mice were treated with ciprofloxacin and metronidazole in their drinking water for 2 weeks followed by either daily supplementation by oral gavage with a probiotic supplement (Bio-25; green), autologous FMT performed after the last day of antibiotics (yellow) or spontaneous recovery (blue). The three groups were killed after 28 d of recovery and a fourth group was killed immediately after antibiotics (magenta). A fifth control group was untreated throughout the 42-d experimental period (grey). From the original experiment, which included 10 mice per group, we randomly selected 5 mice (spanning both cages per group) and performed shotgun metagenomic sequencing and resistome profiling of caecal and distal colon luminal content using ShortBRED and CARD after subsampling to 1.5 M of reads. Results are based on ARG families. **b**, Bray–Curtis dissimilarities. PC1 antibiotics versus control *P* = 0.0317, probiotics versus control *P* = 0.0317, probiotics versus spontaneous *P* = 0.0317. **c**,**d**, Shannon alpha diversity in the caecum lumen (**c**) or distal colon lumen (**d**). The antibiotics group was not included in the distal colon panel because four samples were under the subsampling threshold. **c**, Probiotics versus spontaneous recovery *P* = 0.0317; probiotics versus control *P* = 0.0317; control versus antibiotics *P* = 0.0317. **e**, Abundance of the *vanSD* gene cluster in the different groups and its Spearman correlation with *B. producta* abundance. **f**,**g**, MGEs significantly (*P* < 0.0001) correlated (Spearman) with *vanSD* abundance. **f**, Integrase, *Blautia* sp. YL58. **g**, IS-10 family transposase, *B. producta*. *P < 0.05; ***P < 0.001; ****P < 0.0001, two-sided Mann–Whitney *U*-test. The horizontal lines represent the median and the whiskers represent the 10–90 percentiles. RPKM, reads per kilobase of reference sequence per million sample reads. Source data

These potentially concerning findings raised the possibility that probiotic-associated post-antibiotic resistome expansion constitutes a unique observation stemming from our experimental design or the supplemented probiotic product we utilized. To generalize our findings, we first asked whether they could be replicated in an animal model. We previously reported that similar to humans, probiotic supplementation to antibiotic-treated mice delays microbiome recovery compared to spontaneous recovery or FMT^27^. Resistome profiling of caecal and colonic luminal samples from these mice (Methods and Fig. 5a) indicated that the resistome of antibiotic-naïve mice was indistinguishable from mice receiving post-antibiotics FMT or recovering spontaneously, although it was significantly different than that of mice receiving antibiotics and probiotics (Fig. 5b). Probiotics were associated with higher post-antibiotic resistome alpha diversity in the caecum (Mann–Whitney *U*-test versus naïve *P* = 0.032, versus spontaneous *P* = 0.032, Fig. 5c) but not in luminal samples from the distal colon (Fig. 5d), paralleling our observation in humans that resistome expansion in the GI tract is not reflected in stool samples. After recovery, we detected two ARGs that bloomed significantly and exclusively in the probiotics group (false discovery rate-corrected *P* < 0.005, effect size = 1.7 for both): *axyY*, which encodes a resistance-nodulation-cell division multi-drug efflux pump associated with resistance to cephalosporin, macrolide, fluoroquinolone and aminoglycoside antibiotics, and *vanSD*, a glycopeptide resistance gene cluster that has been reported in vancomycin-resistant isolates of *Enterococcus faecium*. In this study, *vanSD* expansion correlated with the bloom of several *Blautia* species (Spearman’s *P* < 0.0001, *r* = 0.79–0.82 for all), including *Blautia coccoides*, *Blautia* *hominis* and *Blautia producta* (Fig. 5e). Extracting all the reads assigned by ShortBRED to this ARG and aligning them to the National Center for Biotechnology Information (NCBI) non-redundant database using BLASTX indicated that 54.1% of the reads were specifically mapped to *B. producta*. Thus, in two distinct mammalian species, post-antibiotics probiotic supplementation was associated with expansion of the resistome in the GI tract. Interestingly, ARGs associated with vancomycin resistance bloomed in both mice and humans and likely stem from bacterial species that proliferate despite probiotic inhibition of microbiome recovery, rather than from the probiotic supplement itself.

Mobile genetic elements (MGEs) may be involved in the spread of ARGs by horizontal transfer between bacterial strains. To assess the potential for horizontal transfer of the ARGs blooming post-antibiotics and probiotics, we quantified the mobilome in all metagenomic samples using ShortBRED in combination with a curated database of transposases, integrases, recombinases and integrons^34^. We observed that, in mice, *vanSD* abundance in the post-antibiotics probiotics group significantly correlated with different MGEs encoded in several *Blautia* species including *Blautia sp.* YL58 (*r* = 0.94, Fig. 5f) and *B. producta* (*r* = 0.8, Fig. 5g). These correlations suggest that ARG-carrying commensal strains that expand in the post-antibiotics probiotics niche may potentially transfer ARGs horizontally to other commensals or pathogens. These observations merit further studies.

### Comparison to other probiotic supplements and clinical trials

**Extended Data Fig. 6:**
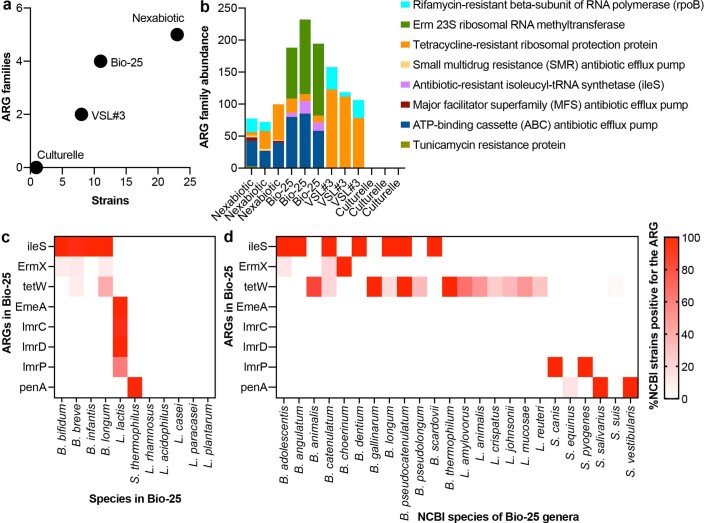
ARG diversity in different probiotics pills. Single-end shotgun metagenomics sequencing was performed on 4 commercially available probiotic products (Bio25, Culturelle, Nexabiotic and VSL#3; 3 pills per product). Resistome profile was quantified using shortBRED, combined with the CARD database: **a**, Abundance of ARG families correlated with the number of strains in the supplement. **b**, Observed ARG families. Presence of Bio25-ARGs (detected by genome assembly, see Methods) in NCBI strain genomes of the Bio-25 species and other species from the same genera: **c**, Percentage of NCBI strains from the Bio-25 species containing an ARG. **d**, Percentage of NCBI strains from other species of the Bio-25 genera containing an ARG. Source data

Whether other probiotic supplements promote resistome expansion, either through ecological effects on the microbiome or rather from encoded resistance genes, is to be determined. As a preliminary exploration of the latter, we profiled ARG diversity among different commercially available oral probiotic supplements (Bio-25, Culturelle, VSL#3 and Nexabiotic; see Methods for the lists of strains). The number of ARG families found in each supplement correlated with the number of strains (Extended Data Fig. 6a). Notably, the various probiotic products displayed different resistome profiles and only tetracycline resistance was shared among multi-strain supplements (Extended Data Fig. 6b). Based on genome assembly, the Bio-25 supplement contained resistance genes to rifamycin, mupirocin, tetracycline, macrolide, streptogramin, lincosamide and β-lactam antibiotics. Notably, presence of the encoding ARGs (*penA*, *lmrD*, *ermX*, *lmrP*, *lmrC*, *emeA*, *ileS* and *tetW*) was not unique to the strains of the Bio-25 supplement since they were also present in a high percentage of strains of the same species (Extended Data Fig. 6c) and in high prevalence in many other species of the same genera (Extended Data Fig. 6d) in the NCBI database. Thus, the presence of ARGs is not uncommon in probiotic strains, although their presence does not necessarily imply phenotypic resistance to antibiotics; other mechanisms (Figs. 4 and 5), rather than horizontal transfer, can mediate probiotic-associated resistome expansion.

**Extended Data Fig. 7:**
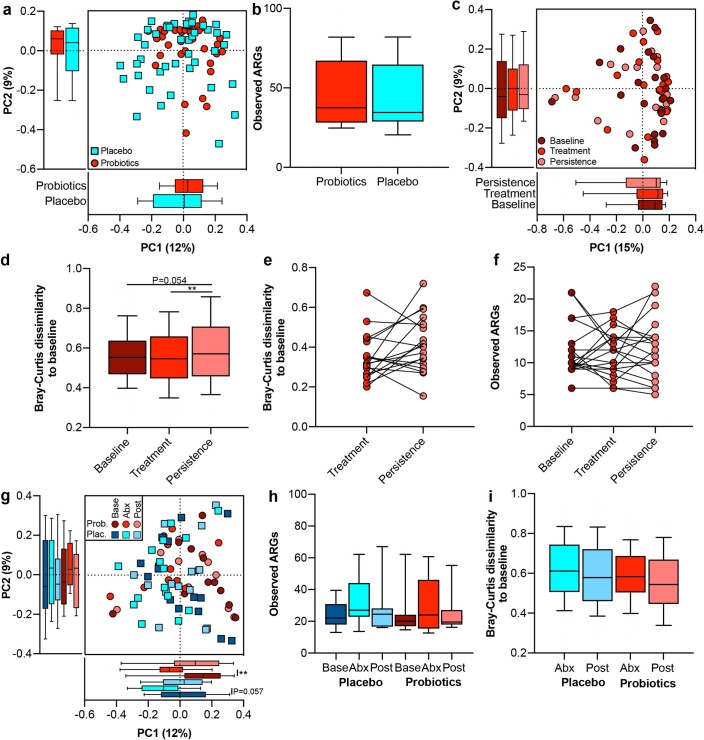
Effect of probiotics and antibiotics on resistome in publicly available datasets. Stool resistome profile of 3 published articles analyzed using shortBRED and CARD database (results based on ARGs): **a-b**, Study of sailors on long sea voyage treated with placebo (blue) or probiotics (samples at the end of the voyage, red); **c-f**, Cohort of 22 participants treated with placebo or probiotics (samples at baseline in dark red, last day of probiotics consumption in red, and 20-week follow-up in light red); **g-i**, Cohort of patients with diabetes treated with placebo (blue) or probiotics (red) after 1-week antibiotics treatment (samples at baseline are dark colored, after antibiotics and after 3-month intervention are light colored). **(a**,**c**,**d**,**e**,**g**,**i**, Beta diversity based on Bray Curtis dissimilarities. **b**,**f**,**h**, Observed ARGs. **d**, Persistence vs. treatment P = 0.0068. **g,** Probiotics baseline vs. antibiotics P = 0.007. **, P < 0.01, two-sided Mann-Whitney. Horizontal lines represent the median, symbols mean, whiskers 10-90 percentiles, error bars SEM. Source data

We next sought to determine whether other probiotics studies reflect our findings. To our knowledge, there are no additional publicly available datasets displaying shotgun metagenomics data from in situ GI tract samples of probiotic-supplemented individuals. Therefore, we analysed data from published studies that utilized stool samples as a proxy of gut-related microbiomes. In the first study, 30-d supplementation with a probiotic containing five *Lactobacillus* and *Bifidobacterium* strains^35^ resulted in no significant resistome differences between probiotics and placebo (Extended Data Fig. 7a) or ARG diversity (Extended Data Fig. 7b). Assessing person-specific effects was not possible due to lack of baseline data. In another study that reported personalized post-supplementation persistence of probiotics^36^ (conceptually similar to our study^26^), there were no global resistome differences between samples collected before and at the end of six months’ supplementation with *Bifidobacterium longum* (Extended Data Fig. 7c). However, the resistome at the end of 20 weeks post-cessation was significantly different than baseline (Mann–Whitney *U*-test *P* = 0.054, Extended Data Fig. 7d) and treatment (*P* = 0.0068, Extended Data Fig. 7d). Per-person analysis suggested that some individuals presented greater resistome dissimilarities between the end of treatment and follow-up, whereas others trended back towards baseline (Extended Data Fig. 7e,f). While personalized differences in colonization permissiveness may underlie the variable effects of probiotics on the resistome, as also observed in our data, the identity of the colonization-permissive individuals was not included in the metadata to verify this association. Finally, to determine whether probiotic-associated expansion of the resistome in antibiotic-treated individuals can be generalized, we analysed the resistome profile of patients with diabetes treated with a 9-strain probiotic for 12 weeks after the administration of antibiotics^37^. Similar to our observations, while antibiotics increased the dissimilarity of the resistome to pre-antibiotics in stool samples (on PC1, placebo Mann–Whitney *U*-test *P* = 0.057, probiotics *P* = 0.007, Extended Data Fig. 7g), antibiotics had no conclusive effect on the number of observed ARGs (Extended Data Fig. 7h) and consequently there were no differences in recovery between the probiotics and placebo groups (Extended Data Fig. 7g–i). While there are several factors that can contribute to the lack of effect of antibiotics on the resistome alpha diversity in this study, our analyses suggest that these may be a result of relying exclusively on stool samples, which may not fully reflect the effect of antibiotics and probiotics on the resistome. Collectively, to further generalize the effects of these interventions on the resistome, additional human studies that include per-participant metagenomic data with direct gut sampling are required.

## Discussion

In this study, we characterized the effects of probiotics and antibiotics on the intestinal reservoir of ARGs by analysing shotgun metagenomic sequencing data from several human cohorts. We report significant differences in the number and type of ARGs present in stool to those observed in luminal aspirates and mucosal brushes from the GI tract of healthy, treatment-naïve humans and therefore focused our analysis on in situ endoscopy GI tract samples. We report that supplementation with a commercial probiotic preparation containing commonly used species can reduce the number of ARGs in the lower GI tract; however, this beneficial effect is restricted to a subset of individuals permissive to probiotic colonization. We also report that treatment with antibiotics (ciprofloxacin-metronidazole for 7 d) expands the number of ARGs in the lumen and mucosa of the lower GI tract, which is mitigated by autologous FMT or spontaneous recovery. In contrast, post-antibiotics probiotic supplementation prevents the reduction in ARG quantity in the lower GI tract lumen and further expands the resistome in the lower GI tract mucosa.

These contrasting effects highlight the importance of considering the ecological context in which probiotics are supplemented. When colonization is resisted by the microbiome, probiotics do not elicit an effect on the microbiome and consequently no beneficial effect on the resistome is observed. On the other edge of the spectrum, microbiome ablation by antibiotics supports probiotic colonization; however, in this niche, probiotics have a pronounced effect on the microbiome, inhibiting most members with the exception of several strains that likely carry the expanded ARGs. This effect was ecologically conserved across host species, as similar strains and resistance genes expanded in humans and mice (*Blautia* spp. and vancomycin resistance genes). While in our study the source of the enriched ARGs was the microbiome, rather than the probiotics themselves, we report that ARGs are present in several commercially available probiotic supplements. Thus, in addition to probiotics expanding ARGs-carrying strains in the antibiotic-perturbed gut, the probiotic strains themselves might serve as a reservoir for resistome expansion in the gut. Further studies are required to assess the potential of horizontal transfer of resistance genes from probiotics to commensals and pathogens in the gut. However, the presence of ARGs in probiotic strains calls for better scrutiny of ARG content in probiotic products to prevent potential adverse effects of probiotics on the human resistome.

The extent to which personalized differences in probiotic colonization^26,36,38^ play a role in modulating their clinical efficacy is to be determined. This work suggests that colonization is in fact important to support a beneficial and clinically relevant effect on resistome reduction. This is further supported by the rapid recovery of the resistome from antibiotic-associated expansion after autologous FMT. Compared to allogeneic FMT or probiotics, autologous transplantation offers greater compatibility between host and microbiome and improves the likelihood of successful entrenchment. Further clinical trials are required to optimize and establish the efficacy of this approach.

While the genes that expanded the most in post-antibiotics probiotic-supplemented individuals in our study confer resistance against vancomycin, have been previously demonstrated to horizontally transfer within the human gut^5,39^ and pose a serious health threat^40,41^, further studies are needed to formally prove that these resistance genes can in fact transfer between the expanding strains and other commensals or pathogens and confer phenotypic resistance. Notably, our analysis suggests a significant correlation between the presence of the resistance genes and MGEs, such as transposases and integrases, that could potentially facilitate horizontal transfer of ARGs to commensals or pathogens.

In addition, the observation that persistent resistome disruption is observed (in stool) more than three months after supplementation ceases, suggest that the effects of probiotics on the gut resistome may be persistent and thus increase the chance of horizontal gene transfer events. The persistent post-antibiotics dysbiosis associated with probiotics^27^ may also contribute to ARG persistence since it can reduce the fitness cost of carrying ARGs^42^. Notably, in this work, probiotics were supplemented after antibiotics and not concomitantly to disentangle the effects of probiotics and antibiotics on the gut microbiome. Additional work is required to determine the effect of concomitant administration of antibiotics and probiotics on the gut resistome. The aforementioned limitations notwithstanding, this work raises a potential concern regarding a possible contribution of widely consumed probiotics to the global emergence of AMR. Thus, in parallel to efforts dedicated to deciphering and validating probiotic efficacy with large-scale cohorts^43–46^, safety should also be considered. Additional work with other types of antibiotics and probiotics, longer follow-up periods and in situ sampling of the GI tract after probiotic cessation are required to fully evaluate such putative risk.

## Methods

### Cohort details

The analyses in this work are based on shotgun metagenomic sequences of human gut endoscopy and stool microbiome samples collected as part of our published studies on probiotics^26,27^. Samples were collected from 36 adult males and females as follows: a cohort providing stool samples on 7 sequential days and undergoing endoscopic examination on the last day without any previous intervention (*n* = 15, 46.6% female, mean age 39.73 ± 14.88 years, mean body mass index 22.71 ± 3.76 kg m^−^^2^). Of these, a sub-cohort supplemented with a commercially available probiotic supplement (Bio-25, SupHerb) and undergoing endoscopic sampling on day 0 and 21 of supplementation, with stool samples provided before, during and after supplementation (*n* = 10, 40% female, 39.5 ± 15.85 years, mean body mass index 22.05 ± 3.35 kg m^−^^2^). Of these ten individuals, we defined two subsets as permissive/resistant to probiotic colonization using the same definition as in our previous work^26^, based on significant increase in the quantity of the supplemented probiotic strains in the lower GI tract mucosa: a cohort of 21 individuals receiving ciprofloxacin and metronidazole for 7 d, followed by 1 of 3 recovery arms: (1) endoscopic sampling and 28 d of probiotics, with additional endoscopic sampling on day 21 and stool samples collected before, during and after antibiotics/probiotics (*n* = 8, 37.5% female, 28.13 ± 2.42 years, mean body mass index 21.48 ± 1.69 kg m^−^^2^); (2) the same protocol but instead of probiotics, participants received autologous FMT on day 0 (*n* = 6, 50% female, 35.5 ± 8.24 years, mean body mass index 24.9 ± 4.14 kg m^−^^2^); (3) same protocol but no post-antibiotic intervention (*n* = 7, 14.3% female, 36 ± 6.83 years, mean body mass index 23.77 ± 1.95 kg m^−^^2^). Additional information regarding the experimental protocols and cohort data can be found in the published works^26,27^. The human trials were approved by the Tel Aviv Sourasky Medical Center institutional review board (approval nos. TLV-0553-12, TLV-0658-12 and TLV-0196-13) and Weizmann Institute of Science Bioethics and Embryonic Stem Cell Research oversight committee (approval nos. 421-1, 430-1 and 444-1) and were reported to https://clinicaltrials.gov/ (identifiers: NCT03218579 and NCT01922830). Written informed consent was obtained from all participants.

### Probiotic supplements

All probiotics groups received the same supplement (Bio-25, SupHerb), which contained 11 common probiotic strains: *Lactobacillus acidophilus*; *Lactobacillus casei*; *Lactobacillus* *paracasei*; *Lacticaseibacillus* *rhamnosus*; *Lactiplantibacillus plantarum*; *Bifidobacterium bifidum*; *Bifidobacterium breve*; *Bifidobacterium longum* subsp. *longum*; *Bifidobacterium longum* subsp. *infantis*; *Lactococcus lactis*; *and Streptococcus thermophilus*. The quantity and viability of the strains was performed in our published work^26^. We performed shotgun metagenomic sequencing to 18 supplement tablets from different batches. We further performed single-end metagenomic sequencing of three tablets of three additional oral probiotic supplements: Culturelle (*L. rhamnosus* GG); VSL#3 (*Lactobacillus helveticus, L. acidophilus, L. paracasei, L. plantarum, B. breve, B. longum* subsp. *longum, B. longum* subsp. *infantis, S. thermophilus*) and Nexabiotic (*Saccharomyces boulardii*, *S. thermophilus,* *Lactobacillus* *delbrueckii, L. rhamnosus, L. plantarum, L. acidophilus, L. casei, L. helveticus, Lactobacillus salivarius, L. lactis, L. paracasei,* *Lactobacillus* *brevis,* *Lactobacillus* *gasseri, B. bifidum, B. breve, B. infantis, B. longum, Bacillus subtilis, Bacillus coagulans*). Since samples were sequenced in a single-end configuration, the resistome profile was analysed using the ShortBRED and CARD databases.

### Mice

This work includes newly performed shotgun metagenomic sequencing of intestinal lumen microbiome samples collected from mice in a published study^27^. In this experiment, 8-week-old male C57BL/6J mice (average initial weight 20 g) were purchased from Envigo and allowed to acclimatize to the animal facility environment for 2 weeks before the experiments. All mice were kept at a strict 24 h light–dark cycle, with lights on from 6:00 to 18:00. Every experimental group consisted of two cages per group to control for cage effect (*n* = 5 per cage). For shotgun sequencing, we randomly chose five mice from each group. For antibiotic treatment, mice were given a combination of ciprofloxacin (0.2 g l^−1^; Sigma-Aldrich) and metronidazole (1 g l^−1^; LKT Laboratories) in their drinking water for 2 weeks. For probiotic supplementation, a single tablet (Bio-25, SupHerb) was dissolved in 10 ml of sterile PBS and immediately fed to mice by oral gavage during the dark phase (4 × 10^9^ colony-forming units kg^−1^ day^−1^). For autologous FMT, faecal pellets were collected before antibiotic administration and snap-frozen in liquid nitrogen; during the day of autologous FMT, the pellets from each mouse were separately resuspended in sterile PBS under anaerobic conditions (75% N_2_, 20% CO_2_, 5% H_2_; Coy Laboratory Products), vortexed for 3 min and allowed to settle by gravity for 2 min. Samples were immediately transferred to the animal facility in Hungate anaerobic culture tubes and the supernatant was administered to the mice by oral gavage. On termination of the experiments, the content within the cavity of the caecum or distal colon was extracted and collected for luminal microbiome isolation. Animal studies were approved by and performed according to the ethical guidelines of the Weizmann Institute of Science Institutional Animal Care and Use committee (application no. 29530816-2).

### Whole-genome shotgun sequencing

For shotgun sequencing of mouse samples, Illumina libraries were prepared using a Nextera DNA Sample Prep kit (catalogue no. FC-121-1031; Illumina) according to the manufacturer’s protocol and sequenced on the Illumina NextSeq platform with a read length of 80 base pairs.

### Microbiome composition analysis

Reads were preprocessed with fastp^47^ for adaptor removal and base quality sliding window trimming. Host reads were removed by Bowtie2 v. 2.4.1 (ref. ^48^) using the human (hg37dec_v0.1) or mouse genome reference (C57BL_6NJ). The cleared FASTQ files were subsampled using Seqtk v.1.3-r114 (https://github.com/lh3/seqtk). We carried out the taxonomic assignment of bacterial DNA relying on exact alignment of *k*-mers with Kraken2 v.2.0.9 (ref. ^49^) against the Genome Taxonomy Database release 89 (https://gtdb.ecogenomic.org/). To improve the accuracy of species level classification, we applied Bayesian re-estimation of bacterial abundance with Bracken v.2.5.3 (ref. ^50^).

### Analysis of antibiotic resistance gene content

For ARG quantification, four different pipelines were used. Subsampled quality-controlled reads were processed with ShortBRED v.0.9.5 (ref. ^30^) using CARD v.1.05 (ref. ^29^) as a reference database to define the composition and abundance of ARGs of each sample. This database includes, by expert human curation, the known molecular sequences and mutations conferring resistance to antibiotics with clinical relevance. ARGs are classified into ARG families (genes with similar function) and drug classes (types of antibiotics targeted by ARGs). Subsampled FASTQ files were also processed with ARG-OAP v.2.0 to obtain the annotation of ARG profiles. ARG-OAP v.2.0 provides model-based identification of assembled sequences using SARGfam, a high-quality profile Hidden Markov Model containing profiles of ARG subtypes and including cell number quantification by using the average coverage of essential single-copy marker genes^28^. We used ARG-OAP with default settings. ARG abundances were normalized by cell number. Similarly, each reference sequence was tagged with its functional gene annotation (ARG subtype) and membership within a class of antibiotics targeted by the gene (ARG type). Moreover, to study the sensitivity of ARG quantification methods, two other approaches were used. Subsampled quality-controlled reads were analysed with an alternative deep learning approach, DeepARG, based on a dissimilarity matrix created from all known categories of ARGs, to overcome the high rate of false negatives of a best alignment approach^32^. Finally, we also described the resistome profile with GROOT^33^, combining the variation graph representation of gene sets with a locality-sensitive hashing indexing scheme to allow for fast read classification. The mean sequencing depth was 4,305,780.54 (s.d. = 4,644,960). Several subsampling sizes (1 M, 1.5 M, 2 M, 3 M, 4 M) were tested in all analyses. Comparative analysis using the aforementioned methods highlighted a trade-off between specificity and sensitivity: the ShortBRED algorithm uses protein markers generated against a background protein reference database (for example, UniRef) that could lead to higher specificity compared to other algorithms. By contrast, the ARG-OAP pipeline includes an ARG database with curated and complete ARG sequences, improving the coverage of ARG detection. Due to the unique nature of this study, including stool samples paired with endoscopy samples at relatively low sequencing depth, we selected 1.5 M and 2 M based on the saturation of resistome alpha diversity (Shannon index) and to maintain sufficient sample size and sensitivity for ARG detection and quantification in all comparisons. We employed cross-validation between ARG-OAP and either ShortBRED and CARD or the NCBI non-redundant database.

### Analysis of MGE content

Subsampled quality-controlled FASTQ files were processed with ShortBRED v.0.9.5 using a reference database of MGEs (transposases, integrases, recombinases and integrons) curated by NanoARG^34^.

### Correlation analysis

ARG and MGE abundances were systematically correlated with species abundances using linear models. Benjamini–Hochberg correction was used for multiple hypothesis testing.

### Detection of ARG types in Bio-25 tablets

Reads from three paired-end sequenced Bio-25 tablets were coassembled using SPAdes v.3.14.1 (metagenomic mode)^51^. The repertoire of ARGs present in the assembled contigs was detected using sraX v.1.5 (ref. ^52^) with the ARGminer database v1.1.1 (ref. ^53^).

### Analysis of NCBI genomes

Protein FASTA files from 18,831 strains of the Bio-25 species and other species from the same genera were downloaded from the NCBI. Sequences were aligned to the ARGminer database v.1.1.1 (ref. ^53^) using BLASTP (identity > 85 and length > 60). Only those ARGs previously detected in the assembled contigs were quantified. For each species, the percentage of strains containing an ARG was computed.

### Analysis of published probiotics studies

Samples were downloaded from their respective NCBI BioProjects (PRJNA643353, PRJNA554501 and PRJNA324129). Sequencing reads were preprocessed using fastp^47^ for adaptor removal and base quality sliding window trimming. Host reads were removed by Bowtie2 v.2.4.1 (ref. ^48^) using human (hg37dec_v0.1). The cleared FASTQ files were subsampled using Seqtk. Subsampled quality-controlled reads were processed with ShortBRED v.0.9.5 (ref. ^30^) using CARD v.1.05 (ref. ^29^).

### Statistical analysis

Analyses of alpha and beta diversity were performed in R v.4.0.4 using the phyloseq^54^ v.1.32.0 and vegan^55^ v.2.5-7 packages. ANOSIM based on sample distances was used to test for differences in the community ARG composition. Kruskal–Wallis test with Dunn’s post-hoc test was used for multiple comparisons; two-way ANOVA was used for longitudinal comparisons between groups, with Sidak’s or Dunnett’s post-hoc tests. For two-group comparisons, a two-tailed Wilcoxon (paired) or Mann–Whitney *U*-test (unpaired) test was used.

### Reporting Summary

Further information on research design is available in the Nature Research Reporting Summary linked to this article.

## Supplementary information

Reporting Summary

## Data Availability

All shotgun metagenomic sequencing data analysed in this work can be found in the European Nucleotide Archive (https://www.ebi.ac.uk/ena/browser/home) under accession nos. PRJEB28097 (human and Bio-25 tablets) and PRJEB42567 (mouse and all probiotic tablets). Source data are provided with this paper.

## Source data

Source Data Fig. 1 Statistical source data.
Source Data Fig. 2 Statistical source data.
Source Data Fig. 3 Statistical source data.
Source Data Fig. 4 Statistical source data.
Source Data Fig. 5 Statistical source data.
Source Data Extended Data Fig. 1 Statistical source data.
Source Data Extended Data Fig. 2 Statistical source data.
Source Data Extended Data Fig. 3 Statistical source data.
Source Data Extended Data Fig. 4 Statistical source data.
Source Data Extended Data Fig. 5 Statistical source data.
Source Data Extended Data Fig. 6 Statistical source data.
Source Data Extended Data Fig. 7 Statistical source data.

## Code availability

No custom code was used in the analysis. The following databases were used: *H. sapiens*, GRCh37 (http://hgdownload.soe.ucsc.edu/goldenPath/hg19/bigZips/); *Mus musculus*, C57BL/6J (https://www.ncbi.nlm.nih.gov/genome/52?genome_assembly_id=422183); Genome Taxonomy Database release 89 (https://data.gtdb.ecogenomic.org/releases/); CARD v.1.05 (https://card.mcmaster.ca/download); Database of Mobile Genetic Elements (transposases, integrases, recombinases and integrons), curated by NanoARG v.1.0 (https://bench.cs.vt.edu/nanoarg/#/home); ARGminer database v.1.1.1 (https://bench.cs.vt.edu/argminer/#/database).
