# Supplementary material for: Probiotics impact the antibiotic resistance gene reservoir along the human GI tract in a person-specific and antibiotic-dependent manner
Source: Nat Microbiol. 2021 Jul 5;6(8):1043–54. doi: 10.1038/s41564-021-00920-0 (PMC8318886; doi:10.1038/s41564-021-00920-0)
Supplement: Supplementary file 1 — Reporting Summary [file 41564_2021_920_MOESM1_ESM.pdf]

nature research

Corresponding author(s): Eran Elinav

Last updated by author(s): May 6, 2021

Reporting Summary

Nature Research wishes to improve the reproducibility of the work that we publish. This form provides structure for consistency and transparency in reporting. For further information on Nature Research policies, see our Editorial Policies and the Editorial Policy Checklist.

Statistics

For all statistical analyses, confirm that the following items are present in the figure legend, table legend, main text, or Methods section.

n/a	Confirmed	
☐	☒	The exact sample size (*n*) for each experimental group/condition, given as a discrete number and unit of measurement
☐	☒	A statement on whether measurements were taken from distinct samples or whether the same sample was measured repeatedly
☐	☒	The statistical test(s) used AND whether they are one- or two-sided<br>*Only common tests should be described solely by name; describe more complex techniques in the Methods section.*
☐	☒	A description of all covariates tested
☐	☒	A description of any assumptions or corrections, such as tests of normality and adjustment for multiple comparisons
☐	☒	A full description of the statistical parameters including central tendency (e.g. means) or other basic estimates (e.g. regression coefficient) AND variation (e.g. standard deviation) or associated estimates of uncertainty (e.g. confidence intervals)
☐	☒	For null hypothesis testing, the test statistic (e.g. *F*, *t*, *r*) with confidence intervals, effect sizes, degrees of freedom and *P* value noted<br>*Give P values as exact values whenever suitable.*
☒	☐	For Bayesian analysis, information on the choice of priors and Markov chain Monte Carlo settings
☒	☐	For hierarchical and complex designs, identification of the appropriate level for tests and full reporting of outcomes
☒	☐	Estimates of effect sizes (e.g. Cohen's *d*, Pearson's *r*), indicating how they were calculated

Our web collection on statistics for biologists contains articles on many of the points above.

Software and code

Policy information about availability of computer code

Data collection	No software was used to collect the data.
Data analysis	No custom code was used in the analysis. We used the following software: Fastp (0.20.1) (https://github.com/OpenGene/fastp), Bowtie2 (2.4.1) (https://github.com/BenLangmead/bowtie2), Seqtk (1.3-r114) (https://github.com/lh3/seqtk), Kraken2 (2.0.9) (https://github.com/DerrickWood/kraken2), Bracken (2.5.3) (https://github.com/jenniferlu717/Bracken), ShortBRED (0.9.5) (https://github.com/biobakery/shortbred), DeepARG (2.0) (https://github.com/gaarangoa/deeparg2.0), ARGs-OAP (2.0) (https://github.com/biofuture/Ublastx_stageone),, GROOT (1.1.2) (https://github.com/will-rowe/groot), Blastp (2.10.1+) (https://blast.ncbi.nlm.nih.gov/Blast.cgi?CMD=Web&PAGE_TYPE=BlastDocs&DOC_TYPE=Download), sraX (1.5) (https://github.com/lgpdevtools/sraX), Spades (3.14.1) (https://github.com/ablab/spades), GraphPad Prism (9.1.0) (https://www.graphpad.com/scientific-software/prism/), R (4.0.4), Vegan R package (2.5-7) (https://rdrr.io/cran/vegan/man/vegan-package.html), Phyloseq R package (1.32.0) (http://www.bioconductor.org/packages/release/bioc/html/phyloseq.html). The following databases were used in the analyses: H. sapiens, GRCh37 (http://hgdownload.soe.ucsc.edu/goldenPath/hg19/bigZips/), Mus Musculus, C57BL_6nJ (https://www.ncbi.nlm.nih.gov/genome/52?genome_assembly_id=422183), Genome Taxonomy Database r89 (https://data.gtdb.ecogenomic.org/releases/), Comprehensive Antibiotic Resistance Database (CARD) (1.05) (https://card.mcmaster.ca/download), Database of mobile genetic elements (transposases, integrases, recombinases and integrons), curated by NanoARG (1.0) (https://bench.cs.vt.edu/nanoarg/#/home), ARGminer database (1.1.1) (https://bench.cs.vt.edu/argminer/#/database).

For manuscripts utilizing custom algorithms or software that are central to the research but not yet described in published literature, software must be made available to editors and reviewers. We strongly encourage code deposition in a community repository (e.g. GitHub). See the Nature Research guidelines for submitting code & software for further information.

Data

Policy information about availability of data

All manuscripts must include a data availability statement. This statement should provide the following information, where applicable:

- Accession codes, unique identifiers, or web links for publicly available datasets
- A list of figures that have associated raw data
- A description of any restrictions on data availability

Data and code availability. All shotgun metagenomics sequencing data analyzed in this work can be found in the European Nucleotide Archive (https://www.ebi.ac.uk/ena/browser/home) under accession numbers ENA: PRJEB28097 (human and Bio-25 pills) and PRJEB42567 (mouse and all probiotic pills). No custom code was used in the analysis. The following databases were used in the analyses: H. sapiens, GRCh37 (http://hgdownload.soe.ucsc.edu/goldenPath/hg19/bigZips/), Mus Musculus, C57BL_6nJ (https://www.ncbi.nlm.nih.gov/genome/52?genome_assembly_id=422183), Genome Taxonomy Database r89 (https://data.gtdb.ecogenomic.org/releases/), Comprehensive Antibiotic Resistance Database (CARD) (1.05) (https://card.mcmaster.ca/download), Database of mobile genetic elements (transposases, integrases, recombinases and integrons), curated by NanoARG (1.0) (https://bench.cs.vt.edu/nanoarg/#/home), ARGminer database (1.1.1) (https://bench.cs.vt.edu/argminer/#/database).

Field-specific reporting

Please select the one below that is the best fit for your research. If you are not sure, read the appropriate sections before making your selection.

☒ Life sciences ☐ Behavioural & social sciences ☐ Ecological, evolutionary & environmental sciences

For a reference copy of the document with all sections, see nature.com/documents/nr-reporting-summary-flat.pdf

Life sciences study design

All studies must disclose on these points even when the disclosure is negative.

Sample size	This work analyzes data from a published clinical trial without adding new participants to the original cohort. No sample size calculations were performed for this work.
Data exclusions	No data were excluded from the analysis.
Replication	The observation that stool samples do not reflect the gastrointestinal resistome in antibiotics-naive individuals was successfully replicated in antibiotics-treated individuals. Probiotics-associated expansion of the gastrointestinal resistome in antibiotics-treated humans was successfully replicated in mice. Reanalysis of additional publicly available data provided some support to the importance of person-specific resistome analysis and direct sampling.
Randomization	This work analyzes data from a published clinical trial without adding new participants to the original cohort. No randomization was performed for this work.
Blinding	This work analyzes data from a published clinical trial without adding new participants to the original cohort. Data analyses were performed on barcoded samples without group identification.

Reporting for specific materials, systems and methods

We require information from authors about some types of materials, experimental systems and methods used in many studies. Here, indicate whether each material, system or method listed is relevant to your study. If you are not sure if a list item applies to your research, read the appropriate section before selecting a response.

Materials & experimental systems

n/a	Involved in the study	
☒	☐	Antibodies
☒	☐	Eukaryotic cell lines
☒	☐	Palaeontology and archaeology
☐	☒	Animals and other organisms
☐	☒	Human research participants
☐	☒	Clinical data
☒	☐	Dual use research of concern

Methods

n/a	Involved in the study	
☒	☐	ChIP-seq
☒	☐	Flow cytometry
☒	☐	MRI-based neuroimaging

Animals and other organisms

Policy information about studies involving animals; ARRIVE guidelines recommended for reporting animal research

Laboratory animals	This work includes newly-performed shotgun metagenomics sequencing of intestinal microbiome DNA samples collected from mice in a published study27. In this experiment, eight-week-old male C57BL/6 mice (average initial weight 20 gr) were purchased from Harlan Envigo.
Wild animals	The study did not involve wild animals.
Field-collected samples	The study did not involve samples collected from the field.
Ethics oversight	Animal studies were approved by and performed according to the ethical guidelines of the Weizmann Institute of Science Institutional Animal Care and Use committee (IACUC), application number 29530816-2.

Note that full information on the approval of the study protocol must also be provided in the manuscript.

Human research participants

Policy information about studies involving human research participants

Population characteristics	This work analyzes data from a published clinical trial without adding new participants to the original cohort. The original cohort was comprised of 50 men and women aged 18-70.
Recruitment	This work analyzes data from a published clinical trial without adding new participants to the original cohort.
Ethics oversight	The human MUSPIC trials were approved by the Tel Aviv Sourasky Medical Center Institutional Review Board (IRB approval numbers TLV-0553-12, TLV-0658-12 and TLV-0196-13) and Weizmann Institute of Science Bioethics and Embryonic Stem Cell Research oversight committee (IRB approval numbers 421-1, 430-1 and 444-1).

Note that full information on the approval of the study protocol must also be provided in the manuscript.

Clinical data

Policy information about clinical studies

All manuscripts should comply with the ICMJE guidelines for publication of clinical research and a completed CONSORT checklist must be included with all submissions.

Clinical trial registration	NCT03218579 and NCT01922830 (clinicaltrials.gov)
Study protocol	The study protocol can be found at: https://clinicaltrials.gov/ (Identifiers: NCT03218579 and NCT01922830)
Data collection	This work analyzes data from a published clinical trial without adding new participants to the original cohort. Data were collected at the Tel Aviv Sourasky Medical Center, Tel Aviv, Israel. Participants were recruited between 2014-2018.
Outcomes	This work analyzes data from a published clinical trial without adding new participants to the original cohort. In the original study primary outcomes were probiotics colonization, effect on intestinal microbiome, and on human gut transcriptome, determined by shotgun metagenomics sequencing, RNA sequencing from gut biopsies, and qPCR. There were no additional measured outcomes.
